# onlineFDR: an R package to control the false discovery rate for growing data repositories

**DOI:** 10.1093/bioinformatics/btz191

**Published:** 2019-03-14

**Authors:** David S Robertson, Jan Wildenhain, Adel Javanmard, Natasha A Karp

**Affiliations:** 1 MRC Biostatistics Unit, University of Cambridge, Cambridge, UK; 2 Quantitative Biology, Discovery Sciences, IMED Biotech Unit, AstraZeneca, Cambridge, UK; 3 Department of Data Sciences and Operations, University of Southern California, Los Angeles, CA, USA

## Abstract

**Summary:**

In many areas of biological research, hypotheses are tested in a sequential manner, without having access to future *P*-values or even the number of hypotheses to be tested. A key setting where this online hypothesis testing occurs is in the context of publicly available data repositories, where the family of hypotheses to be tested is continually growing as new data is accumulated over time. Recently, Javanmard and Montanari proposed the first procedures that control the FDR for online hypothesis testing. We present an R package, onlineFDR, which implements these procedures and provides wrapper functions to apply them to a historic dataset or a growing data repository.

**Availability and implementation:**

The R package is freely available through Bioconductor (http://www.bioconductor.org/packages/onlineFDR).

**Supplementary information:**

Supplementary data are available at *Bioinformatics* online.

## 1 Introduction

Multiple hypothesis testing is a common feature of genome bioinformatics and computational biology, and appropriately correcting for this multiplicity is crucial when it comes to making statistical inference from the data. Indeed, uncorrected hypothesis testing has been highlighted as one of the contributing factors to the reproducibility crisis in scientific research (Ioannidis, 2005). The false discovery rate (FDR), which was introduced by Benjamini and Hochberg (1995), has become the error criterion of choice for large-scale multiple hypothesis testing. The FDR is defined as the expected proportion of the discoveries (i.e. rejections) made that are false. To control the FDR, procedures (such as the well-known Benjamini–Hochberg procedure) have been developed which require that all the *P*-values are available to be tested at once.

However, modern data analysis often has a further complexity in that hypotheses are tested sequentially, with the family of hypotheses continually growing due to the temporal accumulation of data. This introduces the challenge of *online* hypothesis testing, where at each step the investigator must decide whether to reject the current null hypothesis without knowing the future *P*-values or even the total number of hypotheses to be tested, but only knowing the historic decisions to date.

This setting occurs in the context of publicly available data repositories, which are becoming increasingly common and important for biological research. Currently, multiple testing in growing data repositories is managed by using a fixed conservative threshold or through the recalculation of significance as new hypotheses are tested. However, the fixed threshold approach fails to adapt to the data, while the recalculation approach can lead to the decisions for an individual hypothesis changing over time.

The online FDR concept is based around hypothesis testing and decisions being made in a sequential manner, with the aim being to control the FDR across the family of hypothesis tests considered. In some biological databases, the family of hypotheses is clearly defined, and a centralized analysis pipeline has been constructed upon which the online FDR method can be implemented. For examples, see the application datasets used in this manuscript. In contrast, in other databases independent research groups may carry out multiple hypothesis testing and generate distinct families of hypothesis tests, and so overall FDR control is not necessarily appropriate.

Javanmard and Montanari (2015, 2018) recently proposed the first procedures that control the FDR for online hypothesis testing, which were the basis for further procedures by Ramdas *et al.* (2017). The R package onlineFDR, available through Bioconductor, implements these procedures and provides wrapper functions to apply them to a historic dataset or a growing data repository.

## 2 Materials and methods

Consider a series of null hypotheses *H*_1_, *H*_2_, *H*_3_, *…* with corresponding *P*-values (*p*_1_, *p*_2_, *p*_3_,* …*). A testing procedure provides a sequence of adjusted significance thresholds *α_i,_*with corresponding decision rules

$$R_{i}=\{\begin{array}{ccc} \;1 & & \text{if}\;\;p_{i}\leq \alpha_{i}\;(\text{reject}\;H_{i})\\ \;0 & & \text{otherwise}\;\;\;\;\;\;\;\;\;\;\;\;\;\;\;\;\; \end{array}$$

A distinction needs to be made between methods appropriate for independent versus dependent *P*-values. As a brief practical example, suppose *p*_1_ corresponds to testing the null hypothesis *H*_1_ that genotype *X* has no association with lean mass, using data *Y* collected on a group of mice. If *p*_2_ corresponds to testing the null hypothesis *H*_2_ that genotype *X* has no association with fat mass using the same data *Y*, then *p*_1_ and *p*_2_ would be dependent due to the association between lean and fat mass for the same mice. However, if instead we tested *H*_2_ using new data *Y’* from a different group of mice, or replaced genotype *X* with an unassociated genotype *X’*, then *p*_1_ and *p*_2_ would be independent.

In the setting of a growing data repository, the online methods have the following baseline assumptions:

1. There is a family of hypothesis tests for which FDR control is required.
2. The hypothesis tests are performed sequentially in time.
3. The *P*-values are all valid and finalized (i.e. will not be changed at a later stage).
4. All of the *P*-values are analysed, and not just the statistically significant *P*-values. An exception is if an orthogonal filter is applied to reduce the dataset size; see Bourgon *et al.* (2010).
5. [For methods requiring independent *P*-values] A different hypothesis is being tested at each step.
6. [For methods requiring independent *P*-values] If the *P*-values come in batches, the ordering within a batch should be random or ordered using independent information.

We now give a high-level overview of the online FDR methods implemented in the package, with full details given in the package vignette (https://www.bioconductor.org/packages/devel/bioc/vignettes/onlineFDR/inst/doc/onlineFDR-vignette.html).

*LOND* stands for ‘significance Levels based On Number of Discoveries’, and provably controls the FDR for independent *P*-values. The values of the adjusted significance thresholds *α_i_* are directly related to the number of discoveries (i.e. rejections) made in the first *i* hypotheses tested. The higher the number of discoveries, the larger the adjusted significance thresholds will be. LOND can be modified to guarantee control FDR under dependent *P*-values, although this can come at the expense of a substantial loss in power.

*LORD* stands for ‘significance Levels based On Recent Discovery’, and also controls the FDR for independent *P*-values. The LORD procedures are examples of generalized alpha-investing rules, and hence have an intuitive interpretation: the procedure starts with an error budget, or alpha-wealth, and there is a price to pay each time a hypothesis is tested. When a new discovery is made, some alpha-wealth is earned back (i.e. there is a ‘return’ on the alpha-wealth invested). The adjusted significance thresholds *α_i_* for LORD procedures thus depend on the alpha-wealth and the times of previous discoveries.

Javanmard and Montanari (2018) presented three versions of LORD, where LORD 1 and 2 provably control the FDR for independent *P*-values, with this only shown empirically for LORD 3. LORD 1 always has smaller significance thresholds (and hence a lower power) than both LORD 2 and LORD 3. The authors also presented an adjusted version of LORD that is valid for dependent *P*-values, but this can lead to a large loss in power. Finally, Ramdas *et al.* (2017) presented a modified version of LORD 2, called LORD++, which always has at least as large significance thresholds (and hence will have an equal or higher power).

*Bonferroni-like procedure*: This controls the FDR for a stream of *P*-values using a Bonferroni-like test. Given a target significance level α, the adjusted significance thresholds are chosen as *α_i_* = *αγ_i_*, where *γ_i_* is a sequence of non-negative numbers that sum to one. This procedure is also valid for dependent *P*-values. Note that for independent *P*-values, the equivalent LOND procedure will always have an equal or higher power.

## 3 Application examples

In practice, using the onlineFDR package on a data repository with a growing family of hypotheses involves the following steps:

1. A dataset is passed to an onlineFDR wrapper function.
2. For each hypothesis test, the adjusted significance threshold *α_i_* is calculated.
3. Using the *P*-values provided and the adjusted significance threshold *α_i_*, an indicator of discoveries *R_i_* is calculated.
4. As the dataset grows, the new larger dataset is passed to the wrapper function, and then *α_i_* and *R_i_* are calculated for the new hypothesis tests (with the previous results remaining the same).

In the Supplementary Material, we apply the procedures to simulated data where the number of false discoveries is a known quantity. This analysis demonstrates that the empirical FDR is correctly controlled over time. We have also applied the procedures to two real-life data repositories (all data and code are available as a Zenodo repository at https://doi.org/10.5281/zenodo.1343578).

The first is from the International Mouse Phenotyping Consortium (IMPC). As described in Karp *et al.* (2017), the IMPC coordinates a large study to functionally annotate every protein coding gene by exploring the impact of the gene knockout on the resulting phenotype for up to 234 traits of interest. Data are uploaded to a public database where phenodeviants are identified using a fixed significance threshold (*P *<* *0.0001). The dataset and resulting family of hypotheses constantly grow as new knockouts are studied. As part of their analysis, Karp *et al.* tested both the role of genotype and the role of sex as a modifier of genotype effect. Hence, the analysis resulted in two sets of *P*-values, one for testing genotype effects and the other for testing sexual dimorphism.

The second dataset, described by Wildenhain *et al.* (2016), contains phenotypic growth data for 240 diverse yeast gene deletion strains grown in the presence of about 5500 unique compounds. This collection has been generated to investigate how small molecule chemical-genetic fingerprints could be used to predict synergistic chemical–chemical combinations that induce lethal phenotypes. Significant phenotypic responses are identified as those with an absolute z-score greater than 4 (or equivalently, *P *<* *0.000032).

**Fig. 1.**
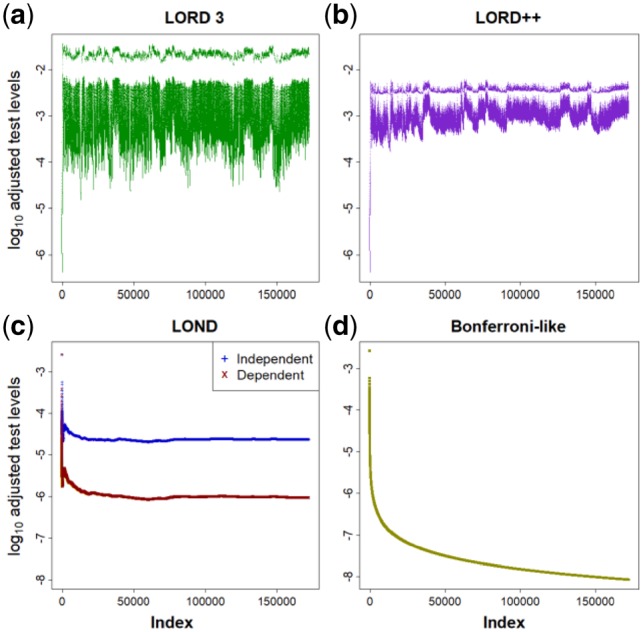
Adjusted significance thresholds on the log10 scale. Applied to genotype effect data from the IMPC dataset, at a FDR level of 5%. (**a**) LORD 3, (**b**) LORD++, (**c**) LOND and (**d**) Bonferroni-like

Visually, we can compare the different procedures by visualizing the adjusted significance thresholds over time (Fig. 1).

We see that for LOND, the adjusted significance thresholds fall away quickly and then remain roughly constant at a very low level. The Bonferroni-like procedure continues to monotonically decrease towards zero and will always have lower significance thresholds than LOND. In contrast, the LORD procedures recover relatively high adjusted significance thresholds when discoveries are made. Visually this can be seen in Figure 1a and b as the adjusted significance thresholds that are elevated due to recent discoveries. This explains why the LORD procedures will typically have a higher power than LOND, which in turn has a higher power than the Bonferroni-like procedures.

Table 1 gives the number of discoveries made by the proposed procedures when applied to the two datasets. As benchmark comparisons, we used the fixed thresholds currently used by the associated databases and the Benjamini and Hochberg (BH) procedure [as well as the adjusted BH that is valid for arbitrary dependencies between *P*-values; see Benjamini and Yekutieli (2001)]. The BH procedure is an offline procedure (i.e. requiring all *P*-values to be available at once), and so in practice could not be applied to a growing data repository, but we include it as a ‘gold-standard’ comparison. The fixed thresholds do not provably control the FDR or adapt to the data over time.

**Table 1.** Number of discoveries made by the online FDR procedures (and benchmark comparisons) for the IMPC and yeast datasets, at a FDR level of 5%

Method	Genotype	SD	Yeast	Method details
Fixed	4158	969	41 767	IMPC < 0.0001
				Yeast < 0.000032
BH	12 907	2084	55 982	Benjamini and Hochberg
LORD 3	9685	1343	53 766	Based on recent discoveries
LORD++	8517	1193	52 352	Modified version of LORD 2
LORD 2	8049	1088	51 864	Based on recent discoveries
LOND	2905	206	44 418	Based on number of discoveries
BH (dep)	4078	315	46 486	BH for arbitrary dependence
LOND (dep)	1475	76	40 325	LOND for dependent *P*-values
LORD (dep)	780	25	36 833	LORD for dependent *P*-values
Bonferroni	795	60	34 363	Bonferroni-like procedure
*N*	172 328	172 328	417 026	

SD, sexual dimorphism; dep, dependent; *N*, total number of *P*-values.

We see that the LORD procedures make more discoveries than the fixed thresholds and (for LORD 2 and LORD++) are recommended as they provably control the FDR. LORD also makes substantially more discoveries than LOND, as seen in Figure 1 above for the IMPC data for example. While LOND makes fewer discoveries than the fixed threshold for the IMPC data, the latter procedure does not guarantee control of the FDR. For the yeast data, the LORD procedures even achieved a similar number of discoveries (93–96%) as the offline BH procedure. Some loss in power is expected when controlling the FDR in an online manner compared to offline procedures. In general, the power of the LORD and LOND procedures tends to increase with the fraction of non-null hypotheses. In the Supplementary Material, we also compare the *sets* of discoveries for the genotype effect data from the IMPC dataset.

Meanwhile, the Bonferroni-like procedure has a relatively low number of discoveries, particularly for the yeast dataset. There is a large drop in the number of discoveries for both LORD and LOND when using methods for dependent *P*-values. The relative power of these procedures compared with the Bonferroni-like one depends on the number of hypothesis tests carried out and on the proportion of true nulls in the dataset; see Robertson and Wason (2018). Further research is required to characterize which dependencies (if any) inflate the FDR when using the LORD and LOND procedures designed for independent *P*-values.

## 4 Conclusion

onlineFDR is an accessible and easy to use R package that controls the FDR for online hypothesis testing. This new tool is particularly useful in allowing bioinformaticians to control for multiplicity in growing data repositories by controlling the FDR across a family of hypotheses. Implementation of this formal framework to manage multiple testing is a substantial improvement over the *ad hoc* methods implemented to date and will help enable robust statistical analyses.

## Supplementary Material

btz191_Supplementary_Data
