# Supplementary material for: onlineFDR: an R package to control the false discovery rate for growing data repositories
Source: Bioinformatics. 2019 Mar 14;35(20):4196–9. doi: 10.1093/bioinformatics/btz191 (PMC6792083; doi:10.1093/bioinformatics/btz191)
Supplement: btz191_Supplementary_Data [file btz191_supplementary_data.pdf]

Supplementary material for "onlineFDR: an R package to control the false discovery rate for growing data repositories"

David S. Robertson[1*], Jan Wildenhain[2], Adel Javanmard[3] and Natasha A. Karp[2]

[1]MRC Biostatistics Unit, University of Cambridge, Cambridge, UK. [2]Quantitative Biology, Discovery Sciences, IMED Biotech Unit, AstraZeneca, Cambridge, UK. [3]Department of Data Sciences and Operations, University of Southern California, CA, USA.

*To whom correspondence should be addressed.

1 Simulation study

In order to compare the different procedures for online FDR control, we used simulated data as described in Javanmard and Montanari (2018). Consider testing *N* hypotheses $H_1, H_2, \ldots, H_N$ concerning the means of normal distributions, where the null hypotheses are $H_i : \theta_j = 0$. The observed test statistics are $Z_j = \theta_j + \varepsilon_j$ where ε_j are independent standard normal random variables. The resulting two-sided *p*-values are given by $p_j = 2(-|Z_j|)$. The mean parameters θ_j are assumed to follow a mixture model:

$$\theta_i = \begin{cases} 0 & \text{with probability } 1-\pi_1 \\ N(0,\sigma^2) & \text{with probability } \pi_1 \end{cases}$$

where $\sigma^2 = 2\log N$. In our simulated dataset, we set $N = 3000$ and $\pi_1 = 0.2$.

Table S1 shows the number of discoveries made by the different procedures, how many of these discoveries are false and the resulting empirical FDR. We see that all methods control the FDR below the nominal 5% level, with the BH and LORD 3 procedures having a similar number of false discoveries and empirical FDR. The LOND, LOND (dep), LORD (dep) and Bonferroni-like procedures make no false discoveries and hence have an empirical FDR of zero, but this is a reflection of their conservatism (as can be seen by the relatively low number of discoveries these procedures make).

Table S1. Number of discoveries made by the online FDR procedures on simulated data, at a target FDR level of 5%.

Method	Number of discoveries	False discoveries	Empirical FDR
Actual	600	–	–
BH	308	12	0.039
LORD 3	272	10	0.037
LORD++	246	6	0.024
LORD 2	240	5	0.021
LOND	184	0	0
BH (dep)	216	1	0.005
LOND (dep)	148	0	0
LORD (dep)	121	0	0
Bonferroni	125	0	0

BH = Benjamini-Hochberg procedure; dep = dependent.

We also consider the cumulative empirical FDR as a function of the number of hypotheses tested. This is shown in Figure S1 below for the LORD 3, LORD++ and LORD 2 procedures. We see that these procedures all control the FDR below the nominal 5% level over time.

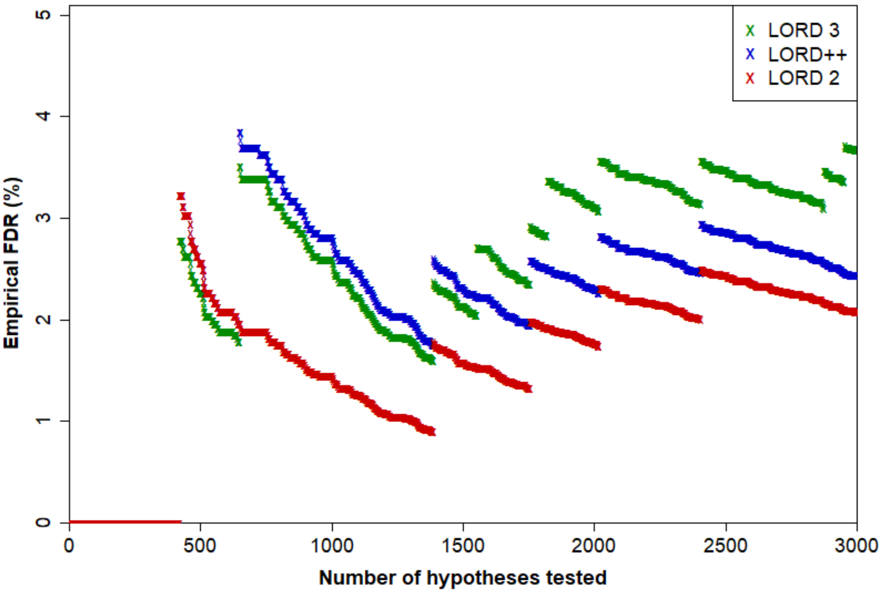

Figure S1. Empirical FDR for the LORD procedures. Applied to simulated data, at a target FDR level of 5%.

2 Application examples – examining sets of discoveries

Table 1 in the paper gives the number of discoveries made by the different methods, but it is also useful to compare the *sets* of discoveries made, in order to give further insight into the properties of the procedures. Table S2 below gives the contingency tables for the discoveries made by the different procedures when applied to the genotype data from the IMPC dataset.

Table S2. Contingency tables for the discoveries made by the online FDR procedures when applied to the genotype data from the IMPC dataset, at a target FDR level of 5%.

		**LORD 3**	
		0	1
**BH**	0	158513	908
	1	4130	8777

		**LORD++**	
		0	1
**BH**	0	159402	19
	1	4409	8498

		**LOND**	
		0	1
**BH**	0	159421	0
	1	10002	2905

		**LORD++**	
		0	1
**LORD 3**	0	161986	657
	1	1825	7860

		**LOND**	
		0	1
**LORD 3**	0	162637	6
	1	6786	2899

		**LORD 2**	
		0	1
**LORD 3**	0	162172	471
	1	2107	7578

		**LOND**	
		0	1
**LORD++**	0	163805	6
	1	5618	2899

		**Bonferroni-like**	
		0	1
**LORD 3**	0	162640	3
	1	8893	792

		**Bonferroni-like**	
		0	1
**LORD++**	0	163808	3
	1	7725	792

		**Bonferroni-like**	
		0	1
**LOND**	0	169423	0
	1	2110	795

We see that the LOND rejections are included in the BH rejections, but this is not the case for LORD 3 (and to a lesser extent LORD++). As further research, it would be interesting (from a statistical perspective) to characterise those genotypes which were not rejected by BH but were by LORD 3. The results also show that the LORD++ rejections are not all included in the LORD 3 rejections, while the LOND rejections are almost all included in those made by LORD 3 and LORD++. Finally, the Bonferroni rejections are always included in the LOND rejections, but there are a few exceptions for the LORD 3 and LORD++ procedures.
